# Histoplasmosis: An epidemiological and clinical update in China, review and a case report

**DOI:** 10.1080/21501203.2023.2259934

**Published:** 2023-09-26

**Authors:** Lihua Chen, Danyang Hu, Congming Zhang, Tianrui Wu, Xiangning Cheng, Ferry Hagen, Hui Zhu, Shuwen Deng

**Affiliations:** a Department of Medicine Laboratory, The Third Xiangya Hospital of Central South University, Changsha, China; b Life Science College, University of Essex, Colchester, UK; c Department of hematology, The Third Xiangya Hospital of Central South University, Changsha, China; d Westerdijk Fungal Biodiversity Institute, Utrecht, The Netherlands; e Department of Gynaecology and Obstetrics, The First Hospital of Suzhou University, Suzhou, China; f Medical Center in The People’s Hospital of SND, Suzhou, China

**Keywords:** Histoplasmosis, *Histoplasma capsulatum*, epidemiology, hemophagocytic lymphohistiocytosis, China

## Abstract

Histoplasmosis is a systemic mycosis caused by the dimorphic fungus in the genus *Histoplasma*. Histoplasmosis is overlooked in China. This study aims to provide an epidemiological and clinical update on histoplasmosis in China by literature review. We reviewed cases of histoplasmosis reported in recent 11 years  and described a case of histoplasmosis-triggered hemophagocytic lymphohistiocytosis (HLH) in an immunocompetent patient. A total of 225 cases of histoplasmosis diagnosed in China between 2012 and 2022 were involved in this study, compared with 300 cases reviewed from 1990 to 2011, an increasing number of cases of histoplasmosis have been diagnosed in the last 11 years. The majority of cases of histoplasmosis were autochthonous cases, mainly from provinces Sichuan (56/225, 24.9%), Hunan (50/225, 22.2%), Guangdong (31/225, 13.8%), and Yunnan (24/225, 10.7%). Higher incidence (52.5%, 53/99) of histoplasmosis occurred in immunocompetent patients which is similar to those from the previous 21 years, and the prevalence of the disease did not vary highly over time. Of note, the number of histoplasmosis cases is increasing, and the geographic distribution is shifting southwards over time. Improved awareness is critically important for informing clinical practice in China.

## 1. Introduction

Histoplasmosis is a worldwide-distributed systemic mycosis caused by *Histoplasma* species, a thermally dimorphic fungi with a specific ecological niche. Its clinical manifestations can range from respiratory infections in immunocompetent individuals to invasive disseminated histoplasmosis in immunosuppressed patients (Benedict and Mody 2016). Histoplasmosis is highly endemic in regions of North, Central, and South America, as well as being reported in parts of Asia and Africa (Bahr et al. 2015; Thompson et al. 2021), but is probably more widespread than previously thought (Antinori 2014). However, the real global burden of histoplasmosis remains unknown due to its non-specific clinical symptoms and radiological findings, whose accurate diagnosis is still challenging (Adenis et al. 2014; Araúz and Papineni 2021).

Histoplasmosis is sorely neglected in China by epidemiologists, researchers, and clinicians, often with fundamental epidemiologic questions still unanswered. Sporadic cases have been reported (Antinori et al. 2021; Ocansey et al. 2022) since the first case of histoplasmosis was reported in 1958 (Lan et al. 2010). A Chinese group has reviewed 300 cases of histoplasmosis reported during 1990 to 2011 in China (Pan et al. 2013) showing a strong association with the Yangtze River flows. Most of the patients had autochthonous infections. A Chinese survey of histoplasmin skin test reactivity among 735 Chinese volunteers with no history of living abroad indicated that the positive rate of histoplasm in skin reaction in Southeast subjects is higher than that in the Northwest which supported the Pan’s study (Zhao et al. 2001). However, epidemiological and clinical data on this disease remain limited due to the lack of comprehensive surveillance for histoplasmosis in China.

This study retrospectively summarises recent 11 years cases of histoplasmosis reported both in English and Chinese about its epidemiological and clinical characteristics, in comparison with previous review data from 1990 to 2011 (Pan et al. 2013), to provide the updated data on prevalence and geographic distribution of histoplasmosis in China, as well as a new case of progressive disseminated histoplasmosis triggered hemophagocytic lymphohistiocytosis in an immunocompetent patient from China.

## 2. Materials and methods

### 2.1. Literature review

A literature search was carried out in databases including PubMed/Medline, Scopus, Web of Science, Embase, and China National Knowledge Infrastructure (CNKI; https://www.cnki.net/). The English and Chinese language (CNKI database) literature between January 2012 and December 2021 was reviewed using search terms “China”, “Chinese”, “histoplasmosis”, and “*Histoplasma* spp.”. The literature search identified 79 published articles about cases of human histoplasmosis including 66 articles in Chinese and 13 in English (Stable 1).

### 2.2. Eligibility criteria for patients’ inclusion

Patients were diagnosed with histoplasmosis based on a global guideline for the diagnosis and management of endemic mycoses (Thompson et al. 2021) in China. Publications were considered only with individual case reports, and case series. The cases included travel-related, and autochthonous cases.

### 2.3. Data collection

The following information was extracted from each article: author, year, language, country of exposure, number of cases, patients’ information (age, gender, occupation, and affected organs), systemic antifungal therapy, and outcomes.

## 3. Result

### 3.1. Case presentation

**Figure 1.**
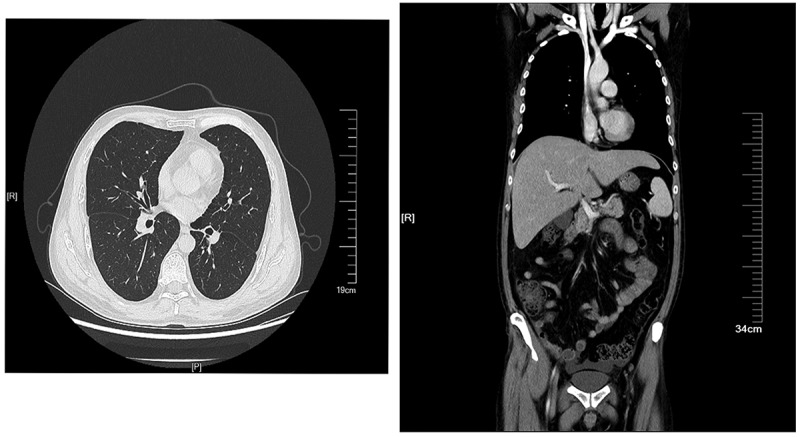
A CT scan of chest and abdomen on the day of admission. A mild inflammation was seen in the lower lobes of bilateral lungs, and the liver was slightly enlarged.

**Figure 2.**
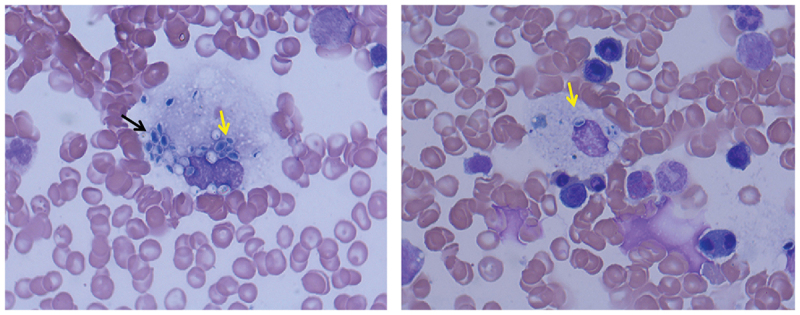
*Histoplasma capsulatum* (numerous, capsulated yeast cells) of phagocyte cells (arrow) in bone marrow smear (Wright’s staining, magnification 1,000×).

**Figure 3.**
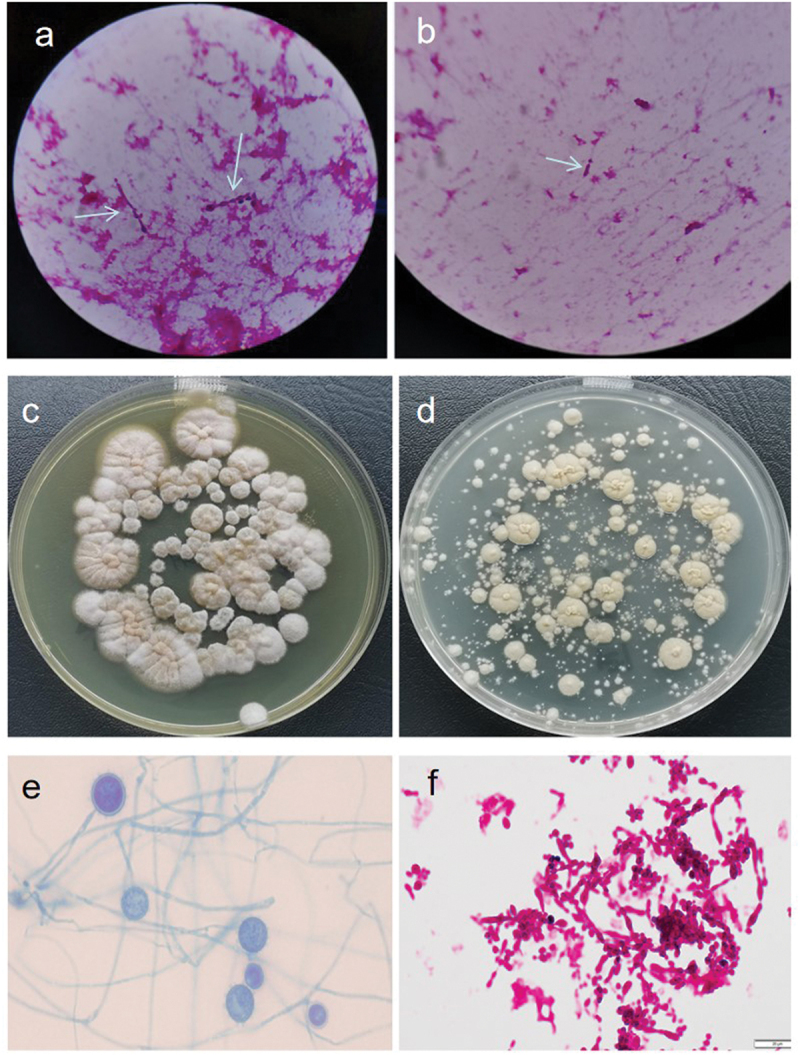
(a, b) Yeast-like spores and hyphae (with an arrow, gram staining, magnification 1,000×) under microscopy with bone marrow cultured at 37 °C in 16 days. (c, d) Colonies grown at 28 °C and 37 °C with bone marrow bone cultured in 16 days; (c) White, short villous and filamentous colonies; (d) Yeast-like colonies. (e, f) Fungal morphology under microscopy by taking colonies, respectively; (e) A filamentous form with tuberculate macroconidia on the septate hyphae (lactic acid phenol cotton blue staining); (f) Yeast-like fungus, budding cells with pseudohyphae (gram staining).

A 44-year-old Chinese male living in Yunnan province was admitted to the emergency department in our hospital on NaN Invalid Date with an intermittent high fever for two months. The patient had worked in Kenya for two years and he started fever after four months back from Kenya. He received empiric treatment with antibiotics at the local hospital for two months, however, fever persisted, while the number of blood platelets reduced to the critical value (<20 × 10^9^/L). Therefore, the patient came to our hospital for further treatment. He denied any medical history. He had experience of eating wild animals (including paguma larvata, pheasant, etc.) during his stay in Kenya. A physical examination at the time of admission revealed a high fever (39.8 °C), hepatosplenomegaly, scleral yellow staining. The patient was suffering from fatigue, progressive weightless >6 kg within two months. His white blood cell (WBC) count was 13.21 × 10^9^/L (83% neutrophils), red blood cell count (RBC) 2.83 × 10^12^/L, haemoglobin (Hb) level 83 g/L and platelet (PLT) count 1 × 10^9^/L. Albumin 17.8 g/L, total bilirubin 55.4 μmol/L, direct bilirubin 40.8 μmol/L. Aspartate and alanine aminotransferase levels were normal. Activated partial thromboplastin time (APTT) and prothrombin time (PT) were prolonged (42.6 seconds and 14.5 seconds, respectively). The serum procalcitonin (PCT) and C-reactive protein (CRP) were elevated (3.342 ng/mL and 71.89 mg/L, respectively). Serologic tests for syphilis, human immunodeficiency virus, hepatitis B and C, and *T*-spot test for tuberculosis were negative. A CT scan of chest and abdomen showed mild inflammation in the lower lobes of bilateral lungs, and the liver was slightly enlarged (Figure 1). The sputum culture showed no positive result. However, Wright-stained bone marrow aspirates revealed intracellular oval or spherical yeast-like cells, which was highly suggestive of *H. capsulatum* (Figure 2), with normal number and classification of megakaryocytes. Although the culturing with bone marrow sample was negative for fungal and bacteria in 5 days incubation, disseminated histoplasmosis was diagnosed based on clinical presentation and visible intracellular yeast-like cells in bone marrow smear. Amphotericin B (30 mg, Qd, i.e.) was administered against fungal infection, meanwhile, cryoprecipitation and platelet transfusion were given to improve the coagulation function. Nevertheless, after 7 days of treatment, the fever persisted, and blood tests showed pancytopenia, WBC 2.22 × 10^9^/L, Hb 63 g/L, PLT 4 × 10^9^/L. APTT and PT prolonged more to 48.6 seconds and 17.2 seconds, respectively. His serum ferritin and sCD25 were elevated to 2,545 μg/L and 35,854 pg/mL, respectively. Then, a second bone marrow puncture was performed, and a bone marrow examination revealed lots of histiocytes phagocytosing red blood cells, erythroblasts, and platelets along with many intrahistiocytic, and extracellular *H. capsulatum* which indicated HLH secondary to histoplasmosis. HLH was diagnosed. However, the culture of the bone marrow aspirate provided negative results after 16 days of incubation. We did a routine check by smear with a negative culture specimen. Interestingly, yeast-like cells and hyphae were seen in the smears (Figure 3a-b). which were compatible with *Histoplasma* spp., then we inoculated the culture specimen on the Sabouraud Dextrose Agar (SDA), incubated at 28 °C and 37 °C, respectively, in 9 days later, it appeared cottony white colonies under culture condition at 28 °C and a yeast-like colony at 37 °C (Figure 3c-d). Microscopic examination showed tuberculate macroconidia and the septated hyphae, and budding cells and pseudohyphae under microscopic (Figure 3e-f). The isolate was identified using PCR by amplifying the ITS1–4 gene, and four sets of specific primers (ARF, H-antigen, OLE, M-antigen) as described previously (Rodrigues et al. 2020), the sequences were blasted in NCBI (https://www.ncbi.nlm.nih.gov/). The isolate was identified as *H. capsulatum*. The sequences were deposited in the GenBank under the accession number ON244035 (ITS), ON478356 (ARF), ON478359 (H-antigen), ON478358 (OLE), ON478357 (M-antigen), which had 99% identity with the *Ajellomyces capsulatum* isolate UAMH 7141. The patient completed the 4-week course of AMB (30 mg, Qd, i.v) and changed to oral itraconazole (200 mg, Bid), which led to the rapid disappearance of fever, with the normal blood test indicators. He was discharged with a prescription of itraconazole 200 mg twice daily for 12 months, and followed up for 1 year, no evidence of relapse was noted.

### 3.2. Summary of the literature

The literature search identified 79 articles about cases of human histoplasmosis both in English and Chinese from China (Figure 3).

A total of 225 cases of histoplasmosis diagnosed in China between year 2012 and 2022 were involved in this study, compared with 300 cases reviewed from year 1990–2011.

### 3.3. Regional distribution

A total of 225 cases of histoplasmosis were diagnosed in China between 2012 and 2022 including our case involved in this study. As shown in Table 1, the 225 patients (162 males, 72.0%) were with a median age of 39.2 years (range 6 months to 75 years old). Only 6.2% (14/225) of the patients were possibly imported cases with travelling history, in contrast, 93.8% (211/225) of the patients had never travelled outside of China, and the most of the cases (71.6%, 161/225) were from four provinces, namely Sichuan (56/225, 24.9%), Hunan (50/225, 22.2%), Guangdong (31/225, 13.8%) and Yunnan (24/225, 10.7%).

**Table 1.** The regional distribution of 225 patients diagnosed as histoplasmosis from 2012 to 2022 in China.

Regional distribution	No. of patients
**Area of suspected Histoplasma exposure**	
South America	11
Mexcica	2
Africa	1
No expoure	211
**Diagnosis place**	
Sichuan	57
Hunan	51
Guangdong	31
Yunnan	27
Zhejiang	9
Hubei	6
Guangxi Zhuang Autonomous Region	5
Shanghai	5
Anhui	4
Jiangxi, Beijing, Shanxi, Henan, Taiwan China	2, respectively
Fujian, Guizhou, Jiangsu, Hainan, Tianjing, Hebei	1, respectively

### 3.4. Clinical characteristics

Among the 225 patients included in this study, 23.5% (53/225) patients were immunocompetent; 9.3% (21/225) were patients with HIV; 4.4% (10/225) with other immunocompromise disease; 6.7% (15/225) with underlying disease, however, nearly half (111/225) of the patients did not describe their immune conditions. Disseminated histoplasmosis (DH) was diagnosed with 178 in 225 (79.2%) cases; the remaining 47 patients presented with chronic pulmonary (41/225, 18.2%), gastrointestinal infection (5/225, 2.2%), and oropharynx infection (1, 0.4%). The most common symptoms were fever (116/225, 51.6%), hepatosplenomegaly (90/225, 40.0%), respiratory symptoms (74/225, 32.9%), digestive tract symptoms (53/225, 23.6%), and lymphadenopathy (41/225, 18.2%). Three cases reported complications with HLH (Table 2).

**Table 2.** The clinical characteristics of 225 patients diagnosed as histoplasmosis from 2012 to 2022 in China.

Clinical characteristics	No. of patients
**Underlying diseases**	
HIV	21
Liver diseases	11
Cancer	7
Organ transplant	2
Autoimmune diseases	1
Diabetes mellitus	4
Healthy	53
Not reported	126
**Site of infection**	
Lung	41
Oropharynx	1
Gastrointestinal	5
Disseminated	178
**Complication**	
HLH	3
Clinical features*	
Fever	116
Splenomegaly/hepatomegaly	105
Respiratory symptoms	74
Digestive tract	53
Lymphadenopathy	41
Pancytopenia	24
Skin	17
Weight loss	18

*Patient may have more than one symptom.

HIV: Human immunodeficiency virus; HLH: Hemophagocytic lymphohistiocytosis.

### 3.5. The diagnosis, treatment, and outcome

The diagnostic work-up varied among the 225 patients: histopathology examination was the main method for diagnosis (94 cases, 41.8%); followed by microscopic visualisation of the fungus with bone marrow smear (62 cases, 27.6%); bone marrow smear + histopathology examination (11 cases, 4.9%); urinary or serum *Histoplasma* antigen (11 cases, 4.9%); culture was available in 34 cases (15.1%); PCR was reported in 8 cases (3.6%) and mNGS was in 2 cases (1.3%). Based on the final diagnosis of the 225 patients, 222 patients were proven as histoplasmosis, and 2 patients were diagnosed as probable histoplasmosis (Table 3).

**Table 3.** The diagnosis, treatment, and outcome of 225 patients diagnosed as histoplasmosis from 2012 to 2022 in China.

Characteristics	No. of patients
**Methods of diagnosis**	
Histology	96
Bone marrow smear	64
Bone marrow smear + histology	17
Histoplasma antigen in urine, serum	11
Culture	16
Bone marrow smear + culture	9
Histology + culture	1
Histology + culture + bone marrow smear	2
Bone marrow smear + culture + PCR	2
Culture + PCR	2
Bone marrow smear + culture + mngs	1
mNGS positive	2
Autopsy	2
**Treatment**	
AMB	66
AMB + itraconazole	46
AMB + FLU	11
Itraconazole	9
AMB + ITR, VOR, CAS	22
Operation + antifungal therapy	10
Not treated	15
Not reported	46
**Outcome**	
Survived	157
Died	10
Not reported	58

AMB: Amphotericin B; ITR: Itraconazole; FLU: Fluconazole; VOR: Voriconazole; CAS: Caspofungin; mNGS: Metagenomics next generation sequencing.

A majority of patients were treated with one of the amphotericin B (AMB) formulations including AMB alone (66/225, 29.3%), AMB + itraconazole (46/225, 20.4%) or + fluconazole (11/225, 4.9%), + other azoles (22/225, 9.8%); 9/225 (4%) patients were treated with itraconazole alone; 10/225 (4.4%) cases with operation combined with antifungals; 14 cases did not receive any treatments. Forty-six patient’s treatment was not recorded. Of the 167 patients whose outcomes were recorded, 10 (6.0%) died (Table 3).

## 4. Discussions

We reported a case of disseminated histoplasmosis-triggered HLH in an immunocompetent patient. The initial clinical presentation, mycological examination, and exposure history in this case were suggestive of histoplasmosis. However, poor clinical response to antifungal therapy, and additional findings such as extreme hyperferritinemia and sCD25, critically helped to establish the diagnosis of HLH secondary to disseminated histoplasmosis. Repeating bone marrow biopsy is often needed, as in most cases hemophagocytosis may not be observed in the initial bone marrow aspirate but will become evidence on follow-up evaluation. Antifungal therapy alone might be sufficient because the symptoms of HLH disappeared completely with the progress of antifungal therapy. Jabr et al. (2019) reviewed 65 cases with histoplasmosis-induced HLH and found that most cases had HIV which exhibited immunodeficiency backgrounds, and most patients were relatively young. However, our case occurred in a relatively young (44 years old) but immunocompetent patient. A literature search in PubMed by the end of September 2017 showed that only four cases worldwide (Jabr et al. 2019) and two cases in China (Lv et al. 2021; Song et al. 2021) had been reported the HLH secondary to *H. Capsulatum* in immunocompetent patients. Therefore, histoplasmosis-associated HLH among immunocompetent patients is a rare complication.

**Figure 4.**
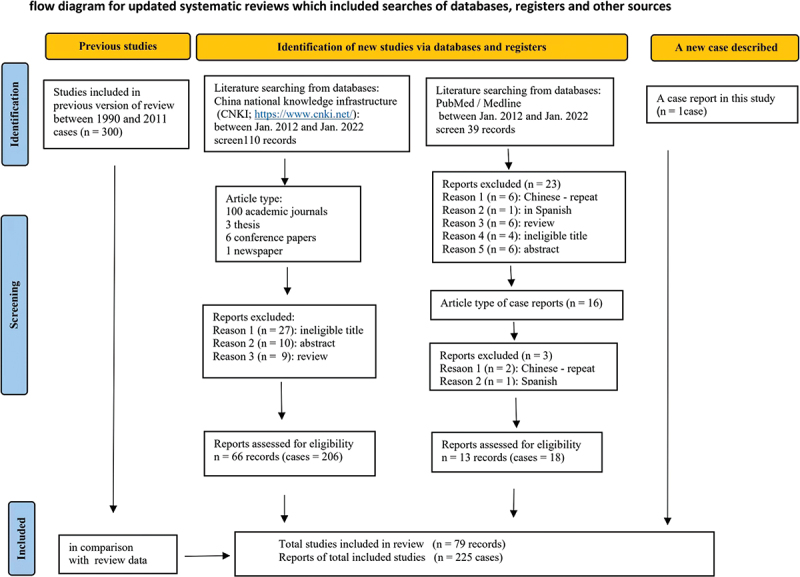
Modified PRISMA flow chart.

**Figure 5.**
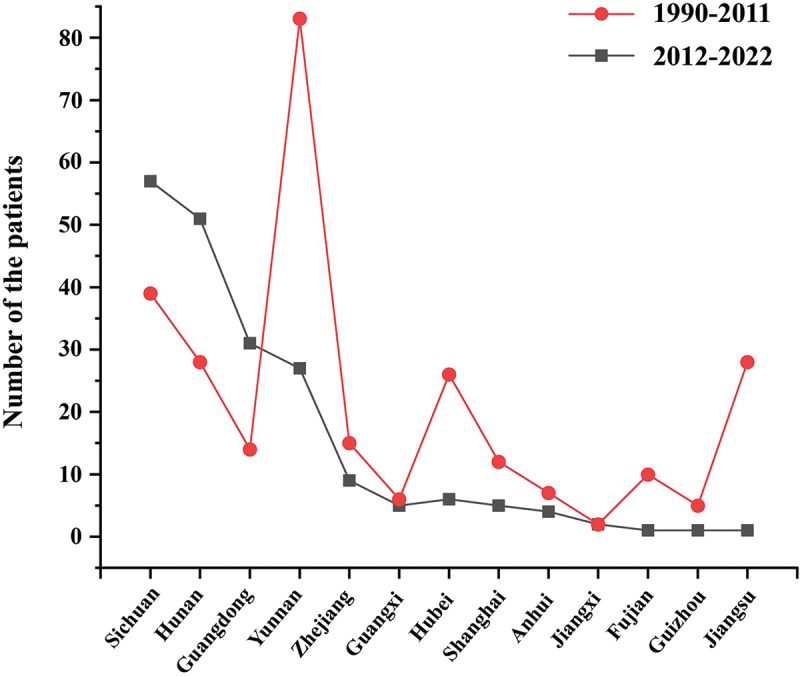
The geographical distribution of histoplasmosis diagnosed in southern China during 2012–2022 compared to those during 1990–2011.

This review summarised 225 cases of histoplasmosis reported from 2012 to 2022 for 11 years, compare with 300 cases reviewed from 1990 to 2011 for 22 years period (Figure 4). An increasing number of cases of histoplasmosis have been diagnosed in the last 11 years. This could be because of the improved laboratory tests and awareness of clinicians in recent years. A review of cases of histoplasmosis from 1990 to 2011 showed a strong association with the regions along the Yangtze River flows and most of the patients had autochthonous infections (Pan et al. 2013). This statement is undoubtedly true but incomplete. Figure 5 shows the geographical distribution of histoplasmosis diagnosed in southern China during 2012–2022 and 1990–2011. Increasing cases in the last 11 years mainly occurred in four provinces, namely, Sichuan (56/225, 24.9%), Hunan (50/225, 22.2%), Guangdong (31/225, 13.8%), and Yunnan (24/225, 10.7%). The number of cases had dropped severely in provinces Yunnan, Hubei, and Jiangsu where are along the Yangtze River flows. Of note, the Guangdong province is in the south, far from the Yangtze River flows where 13.8% of cases were diagnosed. Thus, our results suggest an expanding focus on histoplasmosis to the southern parts of China. Our data also confirmed the skin-test study (Zhao et al. 2001) that its geographic range is much broader than it is often appreciated. The Conditions suitable for *H. capsulatum* have shifted southwards in the past decade, perhaps resulting from a humid and warming climate, anthropogenic land use, and today’s mobile population which in favour for the growth and dispersal of *H. capsulatum*.

Another important message arising from our review is that only 13 cases of histoplasmosis reported the suspected exposure history, 93.8% (211/225) cases of histoplasmosis were not associated with any disclosed history of travel or residence in areas considered to be endemic for the disease and can, therefore, possibly be classified as autochthonous. These findings are in agreement with data from the previous review that 91.3% of the histoplasmosis cases were definitely acquired within China (Pan et al. 2013). This might be due to greater inoculum exposure although most people with histoplasmosis may, in fact, not have clear or memorable exposures to bird or bat droppings.

A higher prevalence of histoplasmosis among immunocompetent individuals (53.5%, 53/99) was observed among 99 cases with clear underly disease descriptions in the literature reviewed (Table 1), which is in line with those (49.1%, 85/173) reported in the previous review (Pan et al. 2013). Similar prevalence of histoplasmosis among patients with HIV (21/99 vs. 38/173) and patients with OIC (25/99 vs. 45/173) were found when compared with previous review (Pan et al. 2013). The conditions suitable for *H. capsulatum* are changing, leading to a corresponding change in epidemiology in the United States as well. One recent retrospective study of 261 cases conducted in the United States found a higher prevalence of disseminated disease among immunocompetent subjects (33%), although it is still an AIDS-defining illness (Franklin et al. 2021). However, Benedict and Mody (2016) reported that histoplasmosis-related hospitalisations have increased over the past decade throughout the United States and are increasingly seen in patients with immunocompromising conditions other than human immunodeficiency virus (HIV)/acquired immunodeficiency syndrome (AIDS).

The patients’ ages ranged between 6 months to 75 years old and a majority of our cases involved males (63.5%, 162/255) and the male-to-female ratio was 2.6:1 which was similar as previous data (2.4:1) (Pan et al. 2013) in China and Europe (2.6:1) (Antinori et al. 2021) but lower than that observed in study from Brazil (4:1) (Almeida et al. 2019). It is in line with a previous review (Pan et al. 2013) that the most common clinical features among the 225 patients were fever (116/225), splenomegaly/hepatomegaly (105/225), and respiratory symptoms (74/225). Gastrointestinal (GI) symptoms and lymphadenopathy have also been noted as frequent symptoms (18%–23.5%) in disseminated diseases. It is worth noting that 23.5% (53/225) of the patients had intestinal histoplasmosis, and 5/225 had localised intestinal histoplasmosis only. Interestingly, HLH was described in two immunocompetent patients in China, which is different from previous studies that HLH was described more frequently among the immunocompromised patients (Nguyen et al. 2020; Antinori et al. 2021).

Histopathology or direct microscopy is still commonly applied in the Lab diagnosis of Chinese cases in both periods. The most diagnosis among 225 cases was based on histopathology and direct microscopy examination (79%, 177/225). Cultures from clinical samples show poor sensitivity (Table 3). Although antigen detection in urine/serum and PCR emerge as valuable adjunctive diagnostic tools, those are not commonly applied in Chinese cases yet due to their expensive cost and are only available in a few laboratories unfortunately.

Amphotericin B (AmB) was as first-line therapy in 86.8% (145/167) of the cases, with good curative effect. It is difficult to evaluate clinical outcomes based on single-case reports and small case series, given that most infected persons are disseminated histoplasmosis, The high proportions of patients who were hospitalised (94%) and died (6%) suggest relatively severe illness for these patients.

However, several limitations should be noted in this review. The data from the literature review does not consider the underreporting of milder cases and underdiagnosed cases, and some incomplete information from the case reports and small case series may have biased our findings. The lack of comprehensive surveillance for these diseases also leaves a gap in our understanding of their true burden in China.

In conclusion, our evidence suggests that the known range of *Histoplasma* endemicity around the Yangtze River flows is shifting. The number of histoplasmosis cases is increasing but the prevalence of the disease has not vary highly over time. It could not estimate the total disease burden yet due to data from the literature review may reflect only a small fraction of the total disease burden while also underestimating the mildest cases.
